# An X-Rays Laboratory

**Published:** 1920-10-09

**Authors:** 


### An X-rays Laboratory.

One of the most interesting deals in the *x*-ray trade that has ever been arranged in this country has just been concluded by X-Rays, Ltd., which has purchased the whole of the *x*-ray and electro-medical business of the High Tension Co., including the plant, stock, goodwill, and many valuable patents. Mr. Mortimer A. Codd, who is well known in connection with his work on high-tension apparatus during the war, and his authorship of a standard work of reference in connection with these instruments, will in future disassociate himself entirely from business, and has entered into an agreement with X-Rays, Ltd., as director of research. This places his excellent laboratory at their services, and should have immense and far-reaching results in the designs of *x*-ray apparatus in the near future.

